# Bioelectronic nose and its application to smell visualization

**DOI:** 10.1186/s13036-016-0041-4

**Published:** 2016-12-13

**Authors:** Hwi Jin Ko, Tai Hyun Park

**Affiliations:** 1 Bio-MAX Institute, Seoul, 151-742 Republic of Korea; 2 School of Chemical and Biological Engineering, Seoul National University, Seoul, 151-742 Republic of Korea; 3 Advanced Institutes of Convergence Technology, Suwon, Gyeonggido 443-270 Republic of Korea

**Keywords:** Olfactory receptor, Odorant, Engineered olfactory cells, Nanovesicles, Bioelectronic nose, Optoelectronic nose, Nano-biotechnology, Smell visualization

## Abstract

There have been many trials to visualize smell using various techniques in order to objectively express the smell because information obtained from the sense of smell in human is very subjective. So far, well-trained experts such as a perfumer, complex and large-scale equipment such as GC-MS, and an electronic nose have played major roles in objectively detecting and recognizing odors. Recently, an optoelectronic nose was developed to achieve this purpose, but some limitations regarding the sensitivity and the number of smells that can be visualized still persist. Since the elucidation of the olfactory mechanism, numerous researches have been accomplished for the development of a sensing device by mimicking human olfactory system. Engineered olfactory cells were constructed to mimic the human olfactory system, and the use of engineered olfactory cells for smell visualization has been attempted with the use of various methods such as calcium imaging, CRE reporter assay, BRET, and membrane potential assay; however, it is not easy to consistently control the condition of cells and it is impossible to detect low odorant concentration. Recently, the bioelectronic nose was developed, and much improved along with the improvement of nano-biotechnology. The bioelectronic nose consists of the following two parts: primary transducer and secondary transducer. Biological materials as a primary transducer improved the selectivity of the sensor, and nanomaterials as a secondary transducer increased the sensitivity. Especially, the bioelectronic noses using various nanomaterials combined with human olfactory receptors or nanovesicles derived from engineered olfactory cells have a potential which can detect almost all of the smells recognized by human because an engineered olfactory cell might be able to express any human olfactory receptor as well as can mimic human olfactory system. Therefore, bioelectronic nose will be a potent tool for smell visualization, but only if two technologies are completed. First, a multi-channel array-sensing system has to be applied for the integration of all of the olfactory receptors into a single chip for mimicking the performance of human nose. Second, the processing technique of the multi-channel system signals should be simultaneously established with the conversion of the signals to visual images. With the use of this latest sensing technology, the realization of a proper smell-visualization technology is expected in the near future.

## Background

The sense of smell plays an important role in human life. Through smelling, the freshness, quality, and taste of food can be assessed, and dangerous materials and situations can be evaluated; moreover, pleasant odors such as a fragrance of flower, perfume, and air freshener can be used for refreshment, and sometimes memories can be recalled. Nevertheless, the exact information of smells cannot be precisely articulated, as the expression of smell is subjective, and the same smell can be recognized differently by different people because smell recognition also depends on personal experience and smelling ability.

More than 100 years ago, scientists tried to describe and classify odors [3, 7, 11, 16, 51, 63], but it proved to be impossible. Recently, various attempts were made to display the smells from a device that was transmitting flavors over the Internet to engineered olfactory cells and optoelectronic noses using metalloporphyrins [31, 44, 68, 69]. But still, it is not easy to visualize smells because there are several limitations, such as a limited number of smells for visualization, a low sensitivity, a low reproducibility, and a complex process, to overcome.

The bioelectronic nose will be a possible solution for the smell-visualization problem. The successful cloning and expression of the human olfactory receptors [32] for the development of the bioelectronic nose and the development of nano-biotechnology have improved the performance of the bioelectronic nose. The bioelectronic nose is constructed with biological materials that have been integrated with nanomaterials such as carbon nanotube, conducting polymer, and graphene [21, 27, 42, 67]. The use of the olfactory receptor as a biological material greatly improved the selectivity of the sensor so that the bioelectronic nose discriminates the difference between single carbon atoms [21], and several nanomaterials extremely enhanced the sensitivity so that the sensor could detect an odorant at a concentration of 0.02 ppt [27].

The sense of smell is importantly used for the daily lives of humans including determination of food freshness, the recalling of memories, and the perception of dangerous materials or dangerous situations like fires; unfortunately, though, anosmia and hyposmia patients cannot be provided with any technological benefits at the present time; therefore, smell visualization is needed for this purpose and it can be achieved by transferring smell information to the general public including people with olfactory problem, who are not experts, by converting the complex electrical signals of bioelectronic nose to any simple visualized form, so that they easily can get or see the smell information without analysis of complex signals. By using olfactory receptors, the bioelectronic nose can also detect a huge variety of smells like the human nose. Thus, it is expected that these properties of the bioelectronic nose will play an important role in smell visualization for many applications, and this review features the potential of the bioelectronic nose as a useful tool for the visualization of smells.

### Human olfaction

The human olfactory system is a sensory system for the smelling of odor molecules and is composed of the area from the olfactory epithelium to the olfactory cortex [53]. Humans can smell and recognize a variety of odorants that are processed by the sense of smell, and this function is known as an olfaction. Odorants reach the mucus-covered olfactory epithelium in the nasal cavity and then bind to the olfactory receptors, which exist in the cilial membrane of the olfactory sensory neurons, and then the odorant information is transferred from the olfactory sensory neurons to the brain. Ion channels are also located in the mucus-covered cilial membranes and play a role in activation of olfactory neurons. Mucus contains odorant-binding proteins that play a major role in the transportation of odor molecules to the olfactory receptor proteins in the aqueous mucus that is facilitated by the solubilization of hydrophobic odor molecules [12, 43]. The binding of odorants to the olfactory receptors induces a conformational change of the olfactory receptor proteins, resulting in a signal transduction through the cAMP pathway or the IP_3_ pathway that is followed by the activation of olfactory neurons by the transportation of ions [6, 18, 47]. The number of the functional olfactory receptors of human is approximately 400, and humans can recognize approximately 10,000 odorants through the combination of these olfactory receptors [33]. The olfactory receptor is the initial part that discriminates and recognizes the odorants in olfaction. Many researchers have tried to utilize the olfactory receptors that are heterologously expressed in various cells for the development of artificial olfactory sensors, as well as for studies on olfaction, and these efforts are becoming fruitful in this research field.

### Artificial olfactory system

#### Engineered olfactory cell

Since the 1990s, many research groups have been trying to figure out olfactory signaling and the deorphanization of olfactory receptors by using the olfactory epithelium tissues that are derived from animals, or by using the animal itself, including a rat [10], frog [48], *C. elegance* [52], and zebra fish [13]. Several limitations, such as difficulties regarding the maintenance of fresh tissues, the attainment of a continuous supply of the same experimental materials from the animals, the analysis of the responses from unidentified olfactory receptors, and the detection and analysis of the signals that are derived from the animals, arose for this kind of approach. To overcome these limitations, engineered olfactory cells were constructed through the heterologous expression of the olfactory receptors in mammalian cells [23, 25], insect cells [45], Xenopus oocyte [66], and yeasts [36]. Especially, the engineered olfactory cells using mammalian cells have been used as a useful tool for deorphanization of the olfactory receptors as well as for the identification of the olfactory signaling [19, 23, 25, 58], since they have a cellular function that is similar to that of the olfactory-receptor neuron cells. To construct engineered olfactory cells, olfactory receptors are connected with the rho-tag sequence, which is a signal sequence of rhodopsin [25], so that they can be expressed on the surface membrane of engineered olfactory cells. The binding of odorants to the olfactory receptors induces a signal cascade that results in an ion influx into the cells. This engineered olfactory cell is a kind of engineered olfactory system in itself because signal transduction is induced by the response of olfactory receptors to specific odorants like an olfactory neuron, and the response can be measured using a variety of methods such as calcium imaging, cAMP response element (CRE) reporter assay, bioluminescence resonance energy transfer (BRET), and membrane potential assay like a recognition by human brain. These measuring tools can visualize the responses of the engineered olfactory cells to odorants; therefore, engineered olfactory cells can be used for smell visualization as well as a primary transducer of the bioelectronic nose.

### Bioelectronic nose

**Fig. 1:**
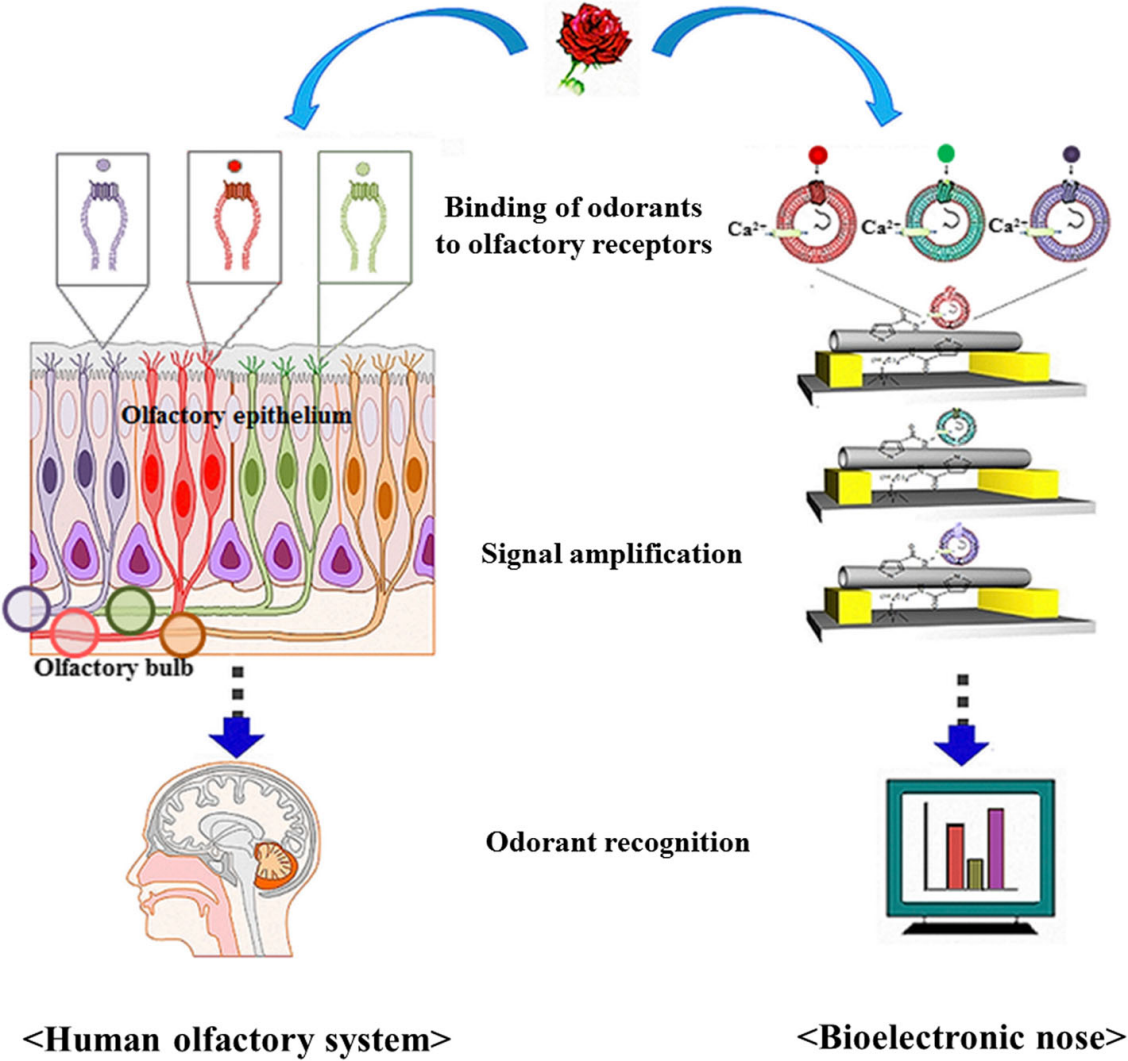
Comparison of the human olfactory system and the bioelectronic nose

The bioelectronic nose was designed on the basis of biological and electrical systems to mimic the human olfactory system (Fig. 1). It consists of two parts. One is the biological recognition part, and the other is the non-biological part. The former plays a major role in the selectivity of the sensor, and the latter plays an important role in the sensitivity of the sensor. For the development of the biosensor, various biological recognition parts such as antibodies, enzymes, aptamers, and sensory receptors have been used as the primary transducer [37, 57, 62]. Following nano-device can be used as a non-biological part in the biosensor: quartz crystal microbalance (QCM) [24, 55, 64], surface plasmon resonance (SPR) [5, 26], nanowire [14], carbon nanotube [21], conducting polymer nanotube [67], and graphene [42]. A combination of biological materials and nano-device enhanced the selectivity and sensitivity of the bioelectronic nose. Especially, the olfactory receptor is a useful biological material for the bioelectronic nose because it can discriminate a target odor molecule from complex odor mixtures and this property considerably enhances the selectivity of the bioelectronic nose.

Recently, various types of bioelectronic noses have been developed. The bioelectronic noses use olfactory receptors, nanovesicles containing olfactory receptors, peptide derived from olfactory receptor proteins as the primary transducer, and use QCM, SPR, and field effect transistor as the secondary transducer. QCM is a crystal sensor for which the piezoelectric effect is used. Various biomaterials, such as lipids, enzymes, and amines are coated on the gold surface of crystal and the adsorption of a specific gas on the coated biomaterials increases the resonant frequency of the crystal [2, 20, 65]. Concentration of specific gas is quantified by the measurement of frequency change [20, 34]. Wu et al. developed an odorant sensing device by using a QCM that is coated with a bullfrog olfactory receptor [64] and Sung et al. constructed a QCM biosensor that is coated with *C. elegans* olfactory receptor ODR10, which is expressed and purified in *E. coli* [55]. For another study, a QCM olfactory sensor was developed using the olfactory receptor I7, which is heterologously expressed in human embryonic kidney (HEK)-293 cells. This sensor showed a specificity and a sensitivity for octanal when it was treated with a variety of odor molecules and various concentrations of octanal [24].

SPR is an optical sensing system for which a surface plasmon wave is used to detect the change of the reflectance on the gold surface caused by binding of biomaterials. SPR is usually used to characterize biomolecular interactions by detecting the amount of ligands as well as the dissociation and association rate of biomolecules [17, 35, 49]. Vidic et al. observed the binding of odorants to olfactory receptors using SPR [60]. In this study, nanosomes containing the rat olfactory receptor I7 and G_αolf_ were derived from yeast co-expressing I7 and G_αolf_. Through odorant stimulation, an SPR that is coated with the nanosomes detected the dissociation of G_αolf_ from the nanosome membranes. The SPR also detected the odorant-induced cellular response [26]. The olfactory receptor I7 was heterologously expressed in HEK-293 cells, and the cells were coated on the gold surface of SPR. When various odorants were applied to the cells, the cells only responded to a specific odorant, octanal, and this cellular response was directly measured by the SPR.

Recently, nanomaterials that are based on field-effect transistors (FETs) have been used for the improvement of the performance of bioelectronic nose. Olfactory receptors are immobilized on the nanotube in bioelectronic nose using the single-walled carbon nanotube (swCNT) and carboxylated polypyrrole nanotube (CPNT) as a nanomaterial. Olfactory receptors commonly contain ionizable cysteine residues which are in a conformational equilibrium state between active and inactive forms and this equilibrium may move to a negatively charged active state by the binding of odorants to olfactory receptors causing the electrostatic perturbation of nanotubes [21, 61]. The single-walled carbon nanotube (swCNT) was combined with a human olfactory receptor that was expressed in *E. coli*, and amylbutyrate was detected at a low concentration of 100 fM with high specificity [21]. In addition, bioelectronic noses were developed for a variety of purposes; for example, nanovesicle-based swCNT-FET sensors were developed for the detection of odor molecules such as heptanal that was produced from lung-cancer patients [30] and geosmin and 2-methylisoborneol for the real-time monitoring of water pollution [54], a CPNT-FET sensor was developed for the detection of odorants in gas phase, and a CNT sensor was immobilized with a canine olfactory receptor for the monitoring of hexanal from spoiled milk [41]. The recently developed bioelectronic noses therefore hold the potential for the detection of a variety of odor molecules like the human olfactory system.

### Smell visualization

#### Smell visualization using optoelectronic nose

**Fig. 2:**
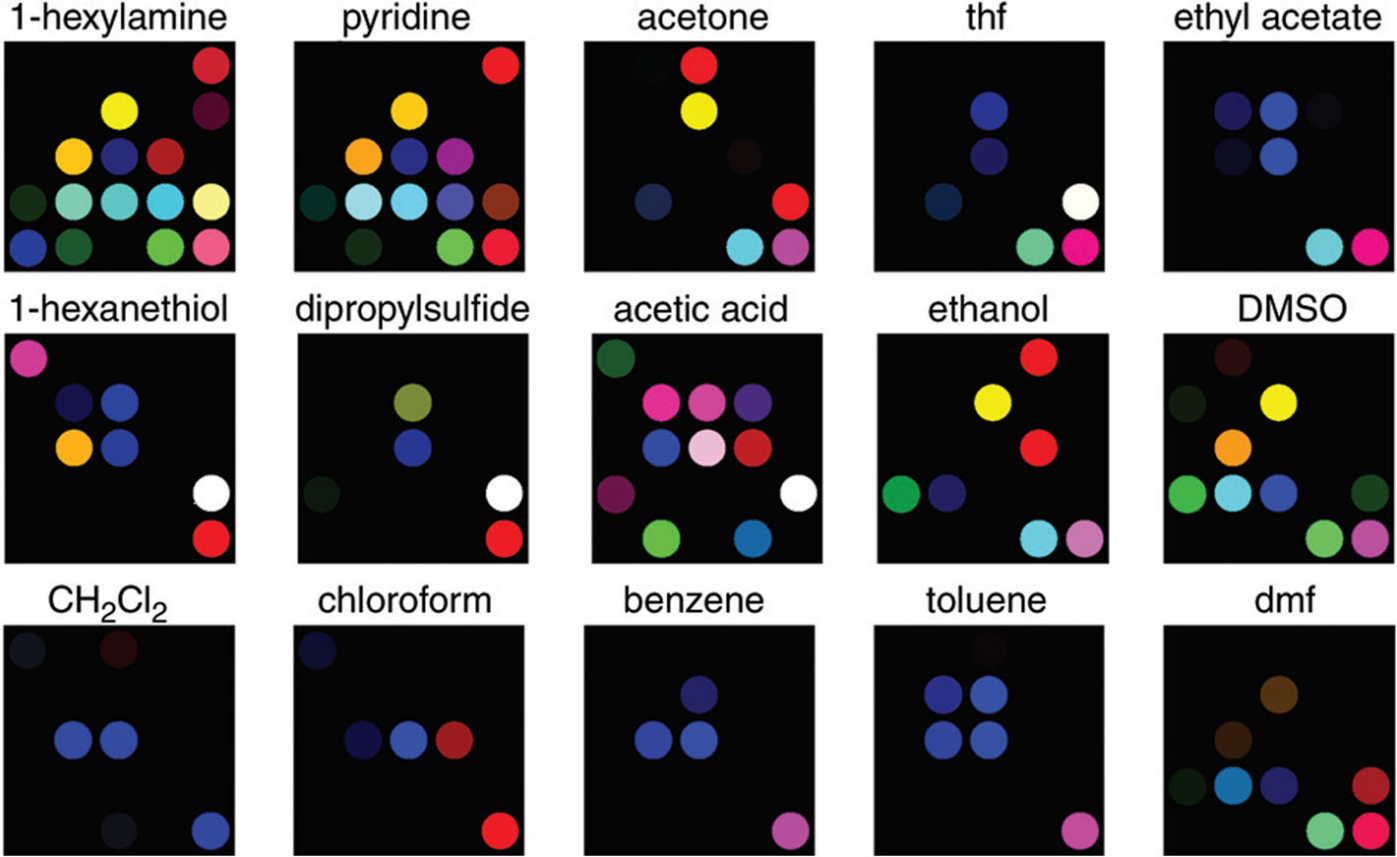
Colorimetric-array responses for some common volatile organic compounds under full vapor pressure at 300 K. Abbreviations: thf _ tetrahydrofuran, DMSO _ dimethylsulfoxide, and dmf _ dimethylformamide. This array system incorporated a series of Zn-substituted, bis-pocketed porphyrins, based on ortho-substitution of the TPP core. Reprinted from [56] with permission from Cambridge University Press)

**Fig. 3:**
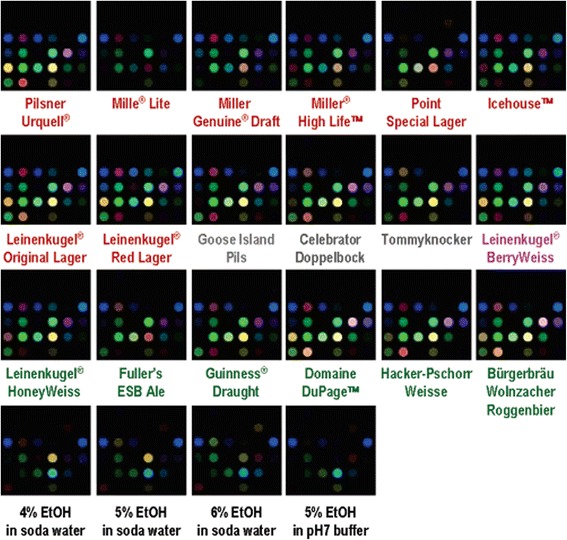
Average color-change profiles of 18 commercial beers and 4 control ethanol solutions for gas-phase analysis. The names are coded with different colors: red, American and Czech lagers; gray, German lagers; green, ales; magenta, specialty; and black, control. For the purposes of an effective visualization, the color range that is shown in these representations is expanded from the RGB values of 4–35 (i.e., 5 bit) to 0–255 (i.e., 8 bit). Color change profiles were obtained from by treatment of sensor arrays with gas-phases of 18 beers. Each beer showed the unique color change profile and these strong responses from beers did not come from ethanol, humidity, or CO_2_. Principal component analysis (PCA) and hierarchical clustering analysis (HCA) were performed for the visual representation of high-dimensional data obtained from color change profiles. Reprinted from [68] with permission from ACS Publications)

For a number of decades, the colorimetric sensor (optoelectronic nose) has been attempted many times, and a variety of materials have been used for odor visualization. Metalloporphyrins have been widely used for the detection of metal-ligating vapors [4, 8, 29, 38, 59]. Optoelectronic noses consisting of metalloporphyrins were developed for the detection of various vapor compounds [56]. These sensor arrays consist of TPP (5, 10, 15, 20-tetraphenylporphyrine) that contains a variety of metal ions and quantified single analytes and identified vapor mixtures. This sensor visualized a variety of volatile organic compounds through a pattern-recognition method (Fig. 2), and colorimetric sensors visualized the quality of a variety of beers (Fig. 3) and toxic gases [31, 68]. The operation of these sensors is simple, and they can classify odors, and the devices can also be potable. But smell visualization using optoelectronic nose is still affected by limitations. In some cases, the device cannot discriminate between a pair of ketones, such as methyl ethyl ketone and 4-heptanone, and the detection limit is sub-ppm [56].

### Smell visualization using engineered olfactory systems

#### Visual representation using engineered olfactory cells

As described above, the signal transduction in engineered olfactory cells is induced by the interaction between odorants and olfactory receptors, and it can be visualized using several methods such as calcium imaging, CRE reporter assay, BRET assay, and membrane potential assay. For the construction of an engineered olfactory cell, human embryonic kidney-293 (HEK-293) cells are transfected with the human olfactory receptor gene, resulting in the expression of olfactory receptors on the surface membrane of HEK-293 cells. When engineered olfactory cells are stimulated by specific odorants, the signal transduction is generated by the activation of the olfactory receptors. The G-proteins are activated, the cAMP levels are increased by the activated G-proteins, and lastly, the cAMP opens the ion channels by allowing an influx of the calcium ions.

**Fig. 4:**
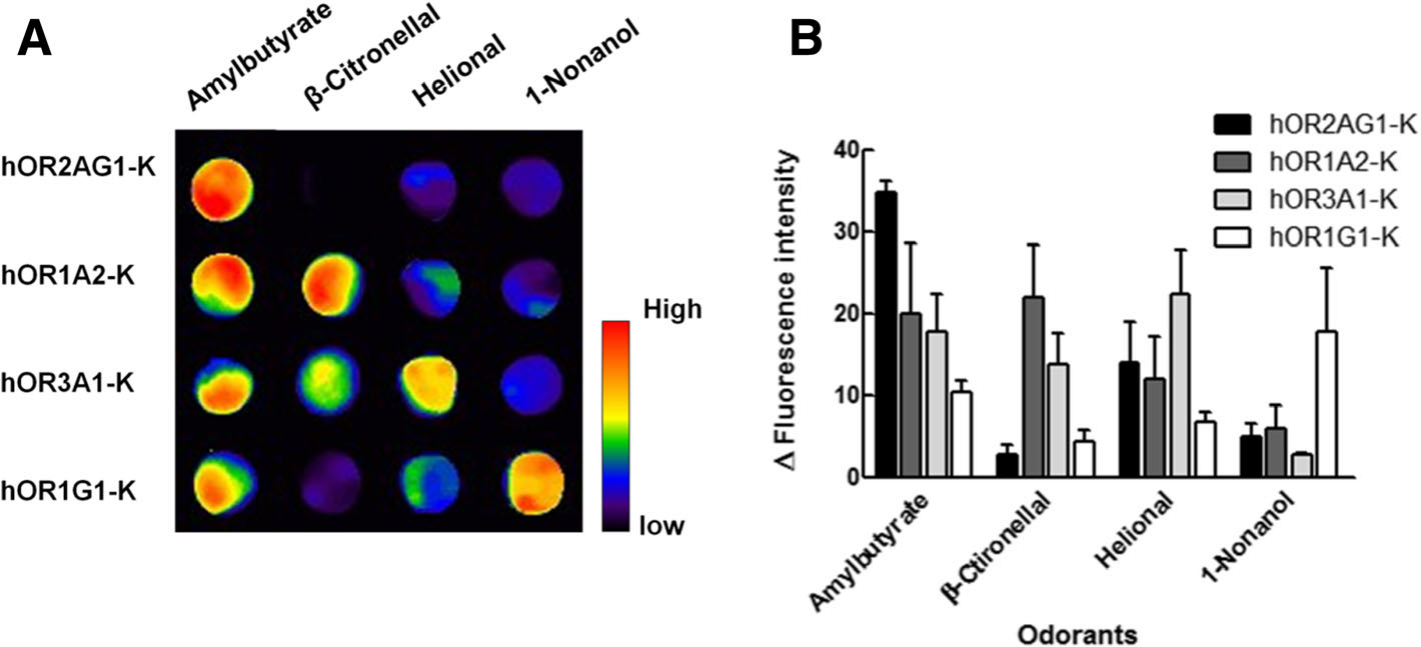
Response of various olfactory receptors to odorants. **a** Odorant response pattern of four different hOR-Ks (hOR2AG1-K, hOR1A2-K, hOR3A1-K, and hOR1G1-K) to various odorants. The hOR-Ks are the fusion proteins of human olfactory receptor and potassium channel. **b** Bar graph of the fluorescence intensity measured from the image scanning data. Error bar represents standard error of mean (*n* = 3). Reprinted from [39] with permission from Elsevier)

The calcium imaging assay detects the increase of the intracellular calcium ions that occurs at the final step of the signal transduction through the use of a calcium-binding fluorescence dye [15, 70]. The CRE reporter assay has been developed as an alternative method, and this assay system made up for the weak points of calcium imaging such as the time-consuming and labor-intensive process [40, 46, 50]. In this system, the activation of the olfactory receptors induces the increase of cAMP level. Luminescence proteins are then expressed through the activation of the CRE promoter that is from the binding of the increased cAMPs to the CRE binding site. Lastly, the change of the luminescence intensity is measured and visualized by a luminometer. The BRET assay is faster than both calcium imaging and CRE reporter assay because it is a protein-based assay whereby the response is immediately detected without a cascade signal transduction. The BRET system was applied to detect the responses of the olfactory receptor ODR-10 to odorants [9]. C-terminus and the third intracellular loop of ODR-10 were connected to a bioluminescent donor and a fluorescent acceptor protein, respectively. The conformational change of ODR-10 is induced by the binding of odorants to ODR-10 and then the donor protein becomes more closely located to the acceptor protein due to the conformational change of the olfactory receptor, resulting in the change of BRET signal. The mechanism of membrane potential assay is simpler when compared with the other methods. Olfactory receptors fused with potassium channels are used in this assay. When an olfactory receptor that is connected with the potassium channel is activated by the binding of a specific odorant, the conformational change of the olfactory receptor is induced and then the potassium channel is opened by this physical change of the olfactory receptor, resulting in an influx of potassium ions through the channel. The change of the membrane potential by the potassium-ion influx can be visualized with membrane potential dye [39]. Figure 4 shows the response of engineered olfactory cells to the stimulation of the odorant that is visualized by the membrane potential assay; thus, engineered olfactory cells can be a tool for the visual representation of smell. Recently, odors in gas phase were detected and visualized using engineered olfactory cells [28], but many challenges still exist for the visualization of smells in gas phase by using cells; moreover, this method is limited to only laboratory environment, whereby the cells should be cultured and constantly maintained for the attainment of reproducible results.

### Visual representation using bioelectronic nose

**Fig. 5:**
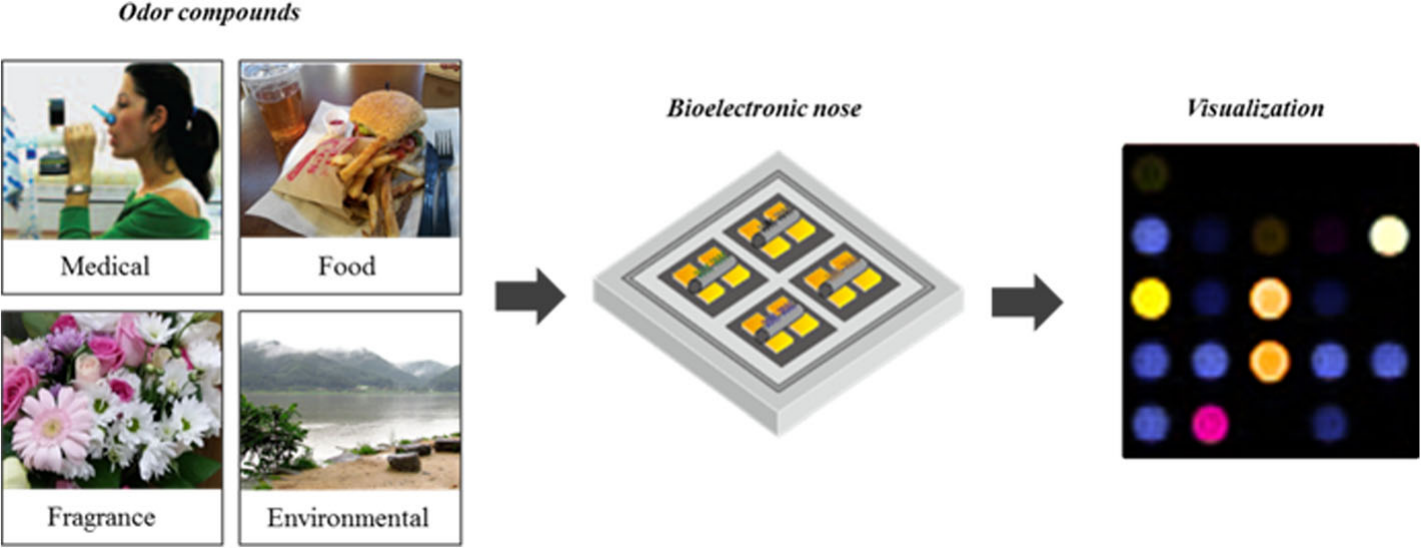
Visualization of odor compounds originating from various sources using a bioelectronic nose. Reprinted from [22] with permission from Elsevier and Nature Publishing Group)

Recently, several types of the bioelectronic nose have been developed for applications in various fields [1, 22, 30, 41, 54] including biomedical and industrial purposes such as disease diagnosis, food quality assessment, and environmental monitoring. In contrast to vision and hearing, smell cannot be exactly described or quantified because the classification and description of smell is quite subjective and abstractive. After the completion of human genome project, sequences of all the human olfactory receptors were identified and the receptors have been used as a representative model set in order to study the interaction of odorants with olfactory receptors as well as the olfactory mechanism through the heterologous expression of them. So, bioelectronic nose using a representative model set of human olfactory receptors may be the best tool mimicking human olfactory system. Therefore, it is expected that visualization of various smells by the bioelectronic nose will be able to provide us with the exact and objective information of smells and these visualized patterns can be used for smell standardization via the encoding of smells like a QR code. The use of the bioelectronic nose for smell visualization is advantageous when it is compared to other engineered olfactory systems and electronic devices for a number of reasons. The bioelectronic nose uses various types of either structures carrying olfactory receptors, olfactory receptors themselves in various environments, or parts of olfactory receptors such as nanovesicle, naodisc, inclusion body, purified protein, and peptide. They are expected to be reproducible, more stable, easier handling than live cells. These receptors enable the bioelectronic nose to stably detect odors in gas phase as well as liquid phase. The bioelectronic nose can detect odor molecules at extremely low concentrations of sub-ppb in gas phase and sub-pM in liquid phase, and it also discriminates smells by mimicking the human olfactory system. It is therefore expected that the outstanding properties of the bioelectronic nose can convert a variety of smells into visual images if the following technologies are successfully combined with the device; One is a multi-channel array sensing system for the integration of all of the olfactory receptors into a single chip for mimicking the performance of human nose, and the other is a multi-signals processing technique for the conversion of the signals into visual images. Thus, bioelectronic nose would be a promising tool for smell visualization with the progress of these techniques (Fig. 5).

## Conclusions

A variety of engineered olfactory systems and sensing techniques with a high selectivity and sensitivity have been developed by mimicking human olfaction system. Various efforts for smell visualization have also been made together with the development of engineered olfactory and sensing systems. The responses of engineered olfactory cells to odors have been converted to visual images using a variety of methods for which the intracellular signals are measured. Colorimetric sensor arrays were also trialed for smell visualization; however, many limitations of these tools still remain. The bioelectronic nose may be an alternative solution for the overcoming of the existing limitations because it is the device that mimicks the human olfactory system most closely, and it can detect smells with a human nose-like function and characteristics. It is expected that the development of smell-visualization tools such as the bioelectronic nose will provides these patients with olfactory benefits, and will also contributes to the variety of industries that are related to food, beverages, and flavor by providing objective olfactory information.

## References

[1] Ahn JH, Lim JH, Park J, Oh EH, Son M, Hong S, Park TH (2015). Screening of target-specific olfactory receptor and development of olfactory biosensor for the assessment of fungal contamination in grain. Sens Actuator B-Chem.

[2] Ameer Q, Adeloju SB (2005). Polypyrrole-based electronic noses for environmental and industrial analysis. Sens Actuator B.

[3] Amoore JE (1963). Stereochemical theory of olfaction. Nature.

[4] Baron AE, Danielson JDS, Gouterman M, Wan JR, Callis JB (1993). Submillisecond response times of oxygen-quenched luninescent coatings. Rev Sci Instrum.

[5] Benilova I, Chegel VI, Ushenin YV, Vidic J, Soldatkin AP, Martelet C, Pajot E, Jaffrezic-Renault N (2008). Stimulation of human olfactory receptor 17-40 with odorants probed by surface plasmon resonance. Eur Biophys J.

[6] Boekhoff I, Breer H (1992). Termination of second messenger signaling in olfaction. Proc Natl Acad Sci U S A.

[7] Brower KR, Schafer R (1975). The recognition of chemical types by odor. The effect of steric hindrance at the functional group. J Chem Educ.

[8] Brunink JAJ, Natale CD, Bungaro F, Davide FAM, D’Amico A, Paolesse R, Boschi T, Faccio M, Ferri G (1996). The application of metalloporphyrins as coating material for quartz microbalance-based chemical sensors. Anal Chim Acta.

[9] Dacres H, Wang J, Leitch V, Horne I, Anderson AR, Trowell SC (2011). Greatly enhanced detection of a volatile ligand at femtomolar levels using bioluminescence resonance energy transfer (BRET). Biosens Bioelectron.

[10] Duchamp-Viret P, Chaput MA, Duchamp A (1999). Odor response properties of rat olfactory receptor neurons. Science.

[11] Dyson GM (1939). The scientific basis of odour. J Soc Chem Ind.

[12] Farbman AI. Cell biology of olfaction. Cambridge University Press;1992.

[13] Friedrich RW, Korsching SI. Combinatorial and chemotopic odorant coding in the Zebrafish olfactory bulb visualized by optical imaging. Neuron. 1997;18(5):737-52. 10.1016/s0896-6273(00)80314-1 9182799

[14] Gao XPA, Zheng G, Lieber CM (2009). Subthreshold regiem has the optimal sensitivity for nanowire FET biosensors. Nano Lett.

[15] Grynkiewicz G, Poenie M, Tsien RY (1985). A new generation of Ca^2+^ indicators with greatly improved fluorescence properties. J Biol Chem.

[16] Henning H (1919). Der Geruch.

[17] Homola J, Yee SS, Gauglitz G (1999). Surface plasmon resonance sensors: review. Sens Actuator B.

[18] Jones ET, Masters SB, Bourne HR, Reed RR (1990). Biochenical characteristics of three stimulatory GTP binding proteins: the large and small forms of Gs and the olfactory specific G protein. Golf J Biol Chem.

[19] Kajiya K, Inaki K, Tanaka M, Haga T, Kataoka H, Touhara K (2001). Molecular bases of odor discrimination: reconstitution of olfactory receptors that recognize overlapping sets of odorants. J Neurosci.

[20] Kanazawa K, Cho NJ (2009). Quartz crystal microbalance as a sensor to characterize macromolecular assembly dynamics. J Sens.

[21] Kim TH, Lee SH, Lee J, Song HS, Oh EH, Park TH, Hong S (2009). Single-carbon-atomic-resolution detection of odorant molecules using a human olfactory receptor-based bioelectronic nose. Adv Mater.

[22] Ko HJ, Lim JH, Oh EH, Park TH, Park TH (2014). Applications and perspectives of bioelectronic nose. Bioelectronic nose.

[23] Ko HJ, Park TH (2006). Dual signal transduction mediated by a single type of olfactory receptor expressed in a heterologous system. Biol Chem.

[24] Ko HJ, Park TH (2005). Piezoelectric olfactory biosensor: ligand specificity and dose-dependence of an olfactory receptor expressed in a heterologous cell system. Biosens Bioelectron.

[25] Krautwurst D, Yau KW, Reed RR (1998). Identification of ligands for olfactory receptors by functional expression of a receptor library. Cell.

[26] Lee SH, Ko HJ, Park TH (2009). Real-time monitoring of odorant-induced cellular reactions using surface plasmon resonance. Biosens Bioelectron.

[27] Lee SH, Kwon OS, Song HS, Park SJ, Sung JH, Jang J, Park TH (2012). Mimicking the human smell sensing mechanism with an artificial nose platform. Biomaterials.

[28] Lee SH, Oh EH, Park TH (2015). Cell-based microfluidic platform for mimicking human olfactory system. Biosens Bioelectron.

[29] Lee WW-S, Wong K-Y, Li X-M, Leung Y-B, Chan C-S, Chan KS (1993). Halogenated platinum porphyrins as sensing materials for luminescence-based oxygen sensors. J Mater Chem.

[30] Lim JH, Park J, Oh EH, Ko HJ, Hong S, Park TH (2014). Nanovesicle-Based Bioelectronic Nose for the Diagnosis of Lung Cancer from Human Blood. Adv Healthc Mater.

[31] Lim SH, Feng L, Kemling W, Musto CJ, Suslick KS (2009). An optoelectronic nose for the detection of toxic gases. Nat Chem.

[32] Mainland JD, Li YR, Zhou T, Liu WLL, Matsunami H (2015). Human olfactory receptor responses to odorants. Sci Data.

[33] Malnic B, Hirono J, Sato T, Buck LB (1999). Combinatorial receptor codes for odors. Cell.

[34] Martin SJ, Granstaff VE, Frye GC (1991). Characterization of a quartz crystal microbalance with simultaneous mass and lipid loading. Anal Chem.

[35] McDonnell JM (2001). Surface plasmon resonance: towards an understanding of the mechanisms of biological molecular recognition. Curr Opin Chem Biol.

[36] Minic J, Persuy M-A, Godel E, Aioun J, Connerton I, Salesse R, Pajot-Augy E (2005). Functional expression of olfactory receptors in yeast and development of a bioassay for odorant screening. FEBS J.

[37] Nambiar S, Yeow JTW (2010). Conductive polymer-based sensors for biomedical applications. Biosens Bioelectron.

[38] Natale CD, Macagnano A, Repole G, Saggio G, D’Amico A, Paolesse R, Boschi T (1998). The exploitation of metalloporphyrins as chemically interactive material in chemical sensors. Mater Sci Eng C.

[39] Oh EH, Lee SH, Ko HJ, Lim JH, Park TH (2015). Coupling of olfactory receptor and ion channel for rapid and sensitive visualization of odorant response. Acta Biomater.

[40] Oh EH, Lee SH, Lee SH, Ko HJ, Park TH (2014). Cell-based high-throughput odorant screening system through visualization on a microwell array. Biosens Bioelectron.

[41] Park J, Lim JH, Jin HJ, Namgung S, Lee SH, Park TH, Hong S (2012). A bioelectronic sensor based on canine olfactory nanovesicle-carbon nanotube hybrid structures for the fast assessment of food quality. Analyst.

[42] Park SJ, Kwon OS, Lee SH, Song HS, Park TH, Jang J (2012). Ultrasensitive flexible graphene based (FET)-type bioelectronic nose. Nano Lett.

[43] Pevsner J, Reed RR, Feinstein PG, Snyder SH (1998). Molecular cloning of odorant-binding protein: member of a ligand carrier family. Science.

[44] Rakow NA, Suslick KS (2000). A colorimetric sensor array for odour visualization. Nature.

[45] Raming K, Krieger J, Strotmann J, Boekhoff I, Kubick S, Baumstark C, Breer H (1993). Cloning and expression of odorant receptors. Nature.

[46] Redmond TM, Ren X, Kubish G, Atkins S, Low S, Uhler MD (2004). Microarray transfection analysis of transcriptional regulation by cAMP-dependent protein kinase. Mole Cell Proteomics.

[47] Reed RR (1992). Signaling pathways in odorant detection. Neuron.

[48] Revial MF, Duchamp A, Holley A. Odour discrimination by frog olfactory receptors: a second study. Chem Senses. 1978;3(1):7-21.

[49] Rich RL, Myszka DG (2008). Survey of the year 2007 commercial optical biosensor literature. J Mol Recognit.

[50] Saito H, Chi Q, Zhuang H, Matsunami H, Mainland JD (2009). Odor coding by a mammalian receptor repertoire. Sci Signal.

[51] Schiffman SS (1974). Physicochemical correlates of olfactory quality. Science.

[52] Sengupta P, Chou JH, Bargmann CI (1996). odr-10 encodes a seven-transmembrane domain olfactory receptor required for responses to the odorant diacetyl. Cell.

[53] Shipley MT, Ennis M, Puche AC, Conn PM (2003). The olfactory system. Neuroscience in Medicine.

[54] Son M, Cho D, Lim JH, Park J, Hong S, Ko HJ, Park TH (2015). Real-time monitoring of geosmin and 2-methylisoborneol, representative odor compounds in water pollution using bioelectronic nose with human-like performance. Biosens Bioelectron.

[55] Sung JH, Ko HJ, Park TH (2006). Piezoelectric biosensor using olfactory receptor protein expressed in *Escherichia coli*. Biosens Bioelectron.

[56] Suslick KS. An optoelectronic nose: “seeing” smells by means of colorimetric sensor arrays. MRS Bull. 2004;720–725. 10.1557/mrs2004.209 15991401

[57] Tombelli S, Minnuni M, Mascini M (2005). Analytical applications of aptamers. Biosens Bioelectron.

[58] Touhara K, Katada S, Nakagawa T, Oka Y. Ligand screening of olfactory receptors. In: Haga, T., Takeda, S, editors. G Protein-coupled Receptors: Structure, Function, and ligand Screening. Boca Raton: CRC Press; 2006. p. 85–109.

[59] Vaughan AA, Baron MG, Narayanaswamy R (1996). Optical ammonia sensing ®lms based on an immobilized metalloporphyrin. Anal Commun.

[60] Vidic JM, Grosclaude J, Persuy M-A, Aioun J, Salesse R, Pajot-Augy E (2006). Quantitative assessment of olfactory receptors activity in immobilized nanosomes: a novel concept for bioelectronic nose. Lab Chip.

[61] Vogel R, Siebert F (2001). Conformations of the active and inactive states of opsin. J Biol Chem.

[62] Wang J (2005). Nanomaterial-based electrochemical biosensors. Analyst.

[63] Wright RH, Serenius RSE (1954). Odour and molecular vibration. II. Raman spectra of substances with the nitrobenzene odour. J Appl Chem.

[64] Wu T (1999). A piezoelectric biosensor as an olfactory receptor for odour detection: electronic nose. Biosens Bioelectron.

[65] Wyszynski B, Somboon P, Nakamoto T (2007). Pegylated lipids as coatings for QCM odor-sensor. Sens Actuator B.

[66] Yasuoka A, Emori Y, Abe K (2000). Addition of signal sequences to the N-termini of olfactory receptor proteins enhances their expression in Xenopus oocyte. Biosci Biotechnol Biochem.

[67] Yoon HS, Lee SH, Kwon OS, Song HS, Oh EH, Park TH, Jang J (2009). Polypyrrole nanotubes conjugated with human olfactory receptors: high-performance transducers for FET-type bioelectronic noses. Angew Chem Int Ed.

[68] Zhang C, Bailey DP, Suslick KS (2006). Colorimetric sensor arrays for the analysis of Beers: A feasibility study. J Agric Food Chem.

[69] Zhang C, Suslick KS (2007). Colorimetric sensor array for soft drink analysis. J Agric Food Chem.

[70] Zufall F, Leinders-Zufall T, Greer CA (2000). Amplification of odor-induced Ca(2+) transients by store-operated Ca(2+) release and its role in olfactory signal transduction. J Neurophysiol.

